# Association of the advanced lung cancer inflammation index and controlling nutritional status score with atrial fibrillation in COPD patients: a multicenter cross-sectional study

**DOI:** 10.3389/fnut.2026.1722288

**Published:** 2026-01-27

**Authors:** Hao Xu, Yanhong Zheng, Tianye Li, Yao Mei, Mengya Yang, Chengshui Chen, Zhidan Hua, Hongjun Zhao

**Affiliations:** 1 Zhejiang Province Engineering Research Center for Endoscope Instruments and Technology Development, Clinical Research Center, Department of Pulmonary and Critical Care Medicine, Quzhou People's Hospital, The Quzhou Affiliated Hospital of Wenzhou Medical University, Quzhou, China; 2 Department of Pulmonary and Critical Care Medicine, Key Laboratory of Interventional Pulmonology of Zhejiang Province, The First Affiliated Hospital of Wenzhou Medical University, Wenzhou, China

**Keywords:** advanced lung cancer inflammation index, atrial fibrillation, chronic obstructive pulmonary disease, controlling nutritional status, cross-sectional study

## Abstract

**Background:**

The coexistence of chronic obstructive pulmonary disease (COPD) and atrial fibrillation (AF) is common and portends a poorer prognosis. This study evaluated whether the Advanced Lung Cancer Inflammation Index (ALI) and Controlling Nutritional Status (CONUT) score—composite biomarkers of inflammation and malnutrition—are associated with AF prevalence in COPD patients.

**Methods:**

This multicenter, cross-sectional study included 1,510 hospitalized patients with COPD. AF was diagnosed according to the European Society of Cardiology (ESC) guidelines, encompassing both a documented clinical history and electrocardiographic evidence. The ALI and CONUT scores were calculated from baseline data. Their independent and combined associations with AF were assessed using multivariate logistic regression, restricted cubic splines (RCS), and analyses of joint groups based on optimal cut-off values. Model performance and improvement were evaluated using the area under the receiver operating characteristic curve (AUC), net reclassification improvement (NRI), integrated discrimination improvement (IDI), and decision curve analysis (DCA). The robustness of the findings was further tested through extensive subgroup and sensitivity analyses.

**Results:**

Among 1,510 patients with COPD, 425 (28.15%) had AF. After comprehensive adjustment for confounders, both a lower ALI and a higher CONUT score were independently associated with increased odds of AF. A nonlinear, L-shaped relationship was identified for ALI (inflection point: 16.09), while CONUT exhibited a linear, positive association. Patients in the combined “low ALI and high CONUT” group had the highest odds of AF (OR = 2.420, 95% CI: 1.721–3.403). The integration of both indices into the baseline model yielded a statistically significant improvement in discriminative power (AUC: 0.842 vs. 0.835, *p* = 0.031), accompanied by substantial reclassification improvement (NRI = 0.273, *p* < 0.001). The findings remained consistent across extensive sensitivity analyses and most clinical subgroups, with a notable interaction observed specifically in patients with pulmonary hypertension.

**Conclusion:**

Lower ALI and higher CONUT scores were significantly associated with a higher prevalence of AF in COPD patients. These readily available composite indices, particularly when used in combination, may aid in identifying patients at increased odds of AF, who could be prioritized for further evaluation.

## 1 Introduction

Chronic obstructive pulmonary disease (COPD), a prevalent chronic respiratory condition affecting over 400 million individuals globally, stands as the third leading cause of mortality worldwide (1–3). Beyond its pulmonary manifestations, COPD is a systemic disorder strongly associated with serious cardiovascular complications. Among these, atrial fibrillation (AF) has garnered increasing clinical attention due to its high prevalence and strong association with worsened outcomes, including increased symptom burden, elevated risks of stroke, heart failure, and all-cause mortality (4–9). For instance, a large population-based study by KuangMing et al. demonstrated that COPD patients face a 2.23-fold higher risk of AF compared to the general population (10), with prevalence rates continuing to climb (11). The early identification of COPD patients at elevated risk for AF is, therefore, a pressing clinical priority.

The pathophysiological link between COPD and AF is multifactorial, with systemic inflammation and malnutrition identified as two pivotal, interconnected mediators. While systemic inflammation in COPD—driven by cytokines such as IL-6 and TNF-*α*—can directly promote atrial fibrillation through oxidative stress, autonomic dysfunction, and atrial structural remodeling (12–15), its role cannot be viewed in isolation. A well-established vicious cycle exists: systemic inflammation promotes a hypermetabolic state, accelerating muscle catabolism and leading to nutritional depletion, which is observed in approximately 25–50% of COPD patients and is independently associated with mortality (16–19). Conversely, malnutrition, characterized by low BMI and hypoalbuminemia, can further exacerbate systemic inflammation and impair immune function (16, 20). This synergistic “inflammation-malnutrition” axis collectively fosters a pro-fibrotic and pro-arrhythmic atrial substrate, significantly increasing susceptibility to AF.

Despite their clinical utility, conventional biomarkers such as C-reactive protein (CRP) for inflammation or albumin for nutrition offer only a unidimensional perspective. They fall short of capturing the integrated burden and complex interplay of the inflammation-malnutrition axis in COPD. This limitation underscores the need for composite indices that can simultaneously reflect these intertwined pathological processes. The Advanced Lung Cancer Inflammation Index (ALI) and the Controlling Nutritional Status (CONUT) score are two such promising tools. ALI, integrating the neutrophil-to-lymphocyte ratio (NLR), body mass index (BMI), and serum albumin, inherently combines inflammatory and nutritional dimensions (21). Similarly, the CONUT score, derived from albumin, total cholesterol, and lymphocyte count, provides a comprehensive nutritional assessment with inherent links to inflammatory status (22). Both indices have demonstrated superior prognostic value over single markers in fields like oncology and cardiology (21, 23), suggesting their potential applicability in the complex landscape of COPD.

However, the specific value of ALI and CONUT, individually or in combination, for AF risk assessment in patients with COPD remains systematically unexplored. To address this evidence gap, this multicenter, cross-sectional study was designed to investigate the associations of ALI and CONUT scores with the prevalence of AF in a COPD population. We hypothesize that these composite indices are significantly associated with AF and that their combined use may offer incremental value in identifying high-risk patients. If validated, these readily available indices could serve as a low-cost, efficient tool for clinicians to flag COPD patients who may benefit from enhanced monitoring and personalized management strategies targeting both inflammation and nutrition, thereby potentially improving clinical outcomes.

## 2 Methods

### 2.1 Study population

This retrospective, multicenter, cross-sectional study was conducted in strict accordance with the STROBE guidelines and approved by the institutional ethics committees following the principles of the Declaration of Helsinki (24). Data were extracted from the electronic medical record systems of the First Affiliated Hospital of Wenzhou Medical University and Quzhou People’s Hospital. During the study period between May 2021 and May 2024, all hospitalized patients diagnosed with both chronic obstructive pulmonary disease (COPD) and atrial fibrillation were consecutively screened and included, while hospitalized patients with COPD without documented atrial fibrillation admitted during the same period were randomly selected as a comparison group, provided that they met the same predefined inclusion and exclusion criteria. The study protocol was approved by the Ethics Committee of the First Affiliated Hospital of Wenzhou Medical University (Approval No.: KY2025-R206) and the Ethics Committee of Quzhou People’s Hospital (Approval No.: 2025–076). Due to the retrospective nature of the study and the use of fully anonymized data, the ethics committees waived the requirement for informed consent from the patients.

**Figure 1:**
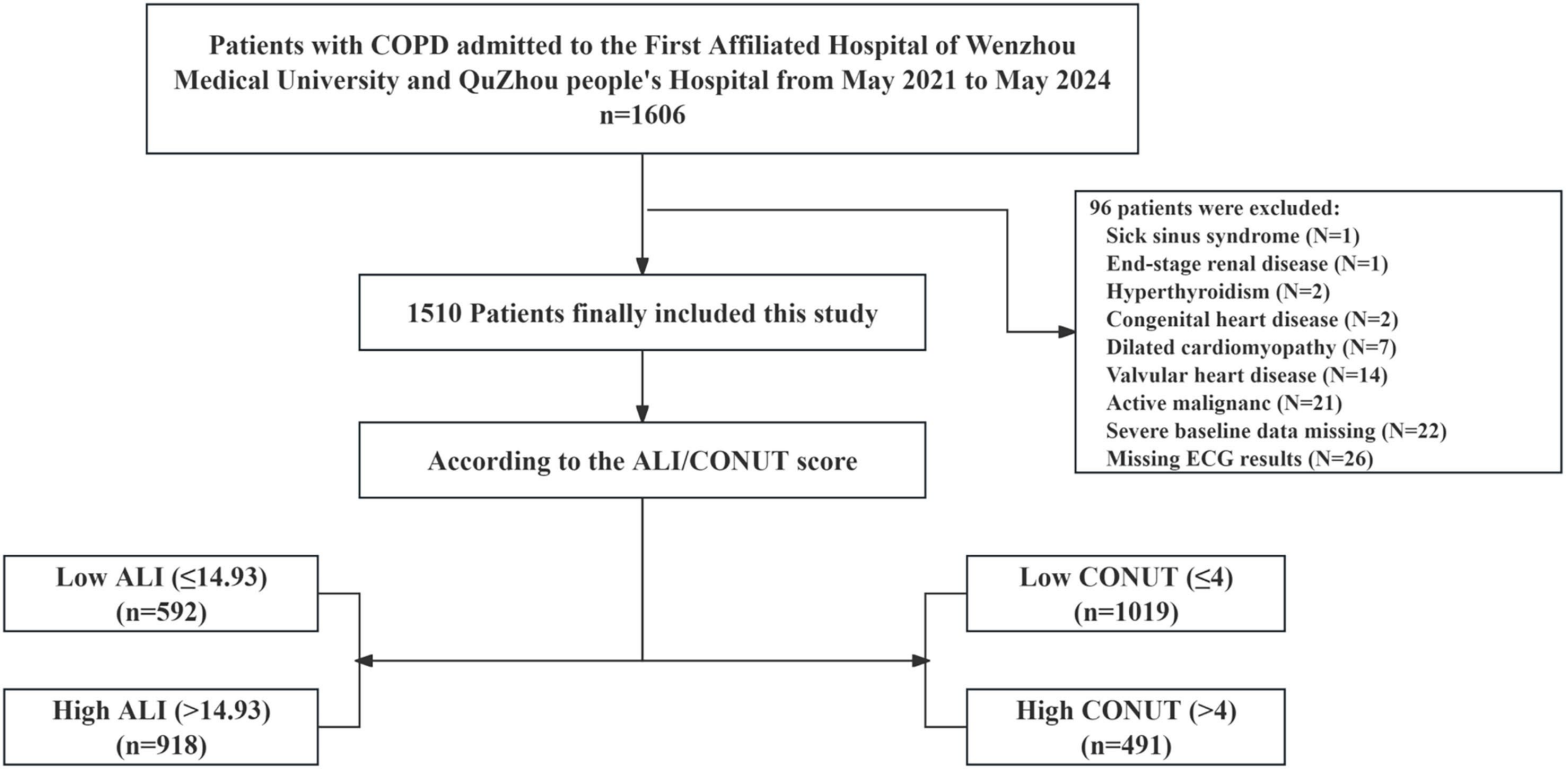
The study’s flow diagram. COPD, chronic obstructive pulmonary disease; ALI, advanced lung cancer inflammation index; CONUT, controlling nutritional status.

The inclusion criteria were as follows: (1) COPD diagnosed in accordance with the 2023 Global Initiative for Chronic Obstructive Lung Disease (GOLD) guidelines (1); (2) age ≥ 18 years; (3) availability of complete baseline clinical data and venous blood laboratory test results collected within 24 h of admission; (4) availability of electrocardiogram (ECG) records—either 12-lead resting ECG or 24-h Holter monitoring—suitable for accurate assessment of cardiac rhythm. To accurately evaluate the relationship between ALI and CONUT scores with the prevalence of AF and to minimize potential confounding factors, we established strict exclusion criteria. Exclusion criteria included: (1) presence of disorders known to be primary causes of atrial fibrillation, such as hyperthyroidism, sick sinus syndrome, preexcitation syndromes, severe valvular heart disease, or congenital heart disease; (2) severe systemic diseases including decompensated liver cirrhosis, active autoimmune diseases, end-stage renal disease (eGFR < 30 mL/min/1.73 m^2^, calculated using the CKD-EPI formula), or active malignancy; (3) missing key variables required for calculating ALI and CONUT scores or missing ECG results; (4) pregnancy or lactation. A total of 1,510 patients were ultimately included for analysis, with the detailed screening process shown in Figure 1.

### 2.2 Data collection, variable definitions, and outcome assessment

Patients were admitted for various clinical reasons, including acute exacerbations of COPD as well as other acute or chronic medical conditions, provided that a diagnosis of COPD was documented at admission. Although a clinical diagnosis of acute exacerbation was recorded when applicable, patients were enrolled based on their admission status, and no systematic differentiation between exacerbation-related and non-exacerbation-related admissions was performed. In addition, detailed information on infection diagnoses or objective measures of acute illness severity at admission was not systematically available and therefore was not incorporated into the analytical models. All data for this study, including demographic characteristics, comorbidities, laboratory parameters, and medication use, were collected at baseline upon hospital admission. The laboratory and anthropometric measurements required to calculate the ALI and CONUT scores were obtained from samples collected and records documented within 24 h of admission.

The primary exposure variables were the ALI and the CONUT score. The ALI was calculated using the following formula (21): ALI = body mass index (BMI, kg/m^2^) × serum albumin (g/dL) / neutrophil-to-lymphocyte ratio (NLR). The CONUT score is a comprehensive nutritional assessment tool based on serum albumin levels, total cholesterol, and total lymphocyte count, with detailed scoring criteria provided in Supplementary Table 1 (22).

The primary outcome of this study was clinical AF. According to the European Society of Cardiology (ESC) guidelines (25, 26), the diagnosis required electrocardiographic (ECG) evidence of a typical AF rhythm lasting ≥30 s, characterized by absolute irregularity in heart rhythm, absence of P waves, and an irregular ventricular rate. This included both a documented history of AF prior to admission and AF newly identified during the index hospitalization based on available ECG or Holter monitoring records. All ECG recordings were independently reviewed by two cardiologists who were blinded to the patients’ ALI and CONUT scores. In cases of disagreement, a final diagnosis was made by a senior electrophysiologist who served as an arbiter.

Covariate data encompassed demographic characteristics (age, sex, BMI, smoking history, alcohol history), comorbidities, laboratory indicators, and medication use during hospitalization. Comorbidities were defined as follows: hypertension was defined as systolic blood pressure ≥140 mmHg and/or diastolic blood pressure ≥90 mmHg or previous diagnosis with ongoing antihypertensive treatment (27); diabetes mellitus as fasting plasma glucose ≥7.0 mmol/L, glycated hemoglobin (HbA1c) ≥ 6.5%, or previous diagnosis with current use of glucose-lowering agents or insulin (28); heart failure based on clinical symptoms, signs, and echocardiographic evidence of left ventricular ejection fraction (LVEF) < 50% (29); coronary artery disease via coronary angiography showing ≥50% stenosis in major vessels, computed tomography angiography, or documented history of myocardial infarction (30); cerebrovascular disease as radiologically confirmed ischemic or hemorrhagic stroke or transient ischemic attack (31); bronchial asthma diagnosed according to the Global Initiative for Asthma (GINA) guidelines (32); and pulmonary hypertension defined as echocardiographically estimated pulmonary artery systolic pressure ≥40 mmHg (33). Laboratory indicators included red blood cell (RBC), white blood cells (WBC), neutrophils (N), lymphocytes (L), monocytes (M), platelets (PLT), alanine aminotransferase (ALT), aspartate aminotransferase (AST), albumin (ALB), blood urea nitrogen (BUN), total cholesterol (TC), high-density lipoprotein cholesterol (HDL-C), low-density lipoprotein cholesterol (LDL-C), and uric acid (UA) levels. Echocardiographic parameters included left ventricular ejection fraction (LVEF). Medication use documentation included in-hospital administration of beta-agonists, anticholinergic agents, aldosterone receptor antagonists, metformin, and beta-blockers.

### 2.3 Data preprocessing

To comprehensively evaluate the association of the ALI and CONUT scores with AF, multiple data processing methods were employed. First, z-score standardization was applied to reduce the influence of extreme values. Simultaneously, to better understand the distribution characteristics of the ALI, patients were categorized into quartiles (Q1-Q4). The CONUT score was stratified into four groups based on clinical standards: normal (0–1 point), mild malnutrition (2–4 points), moderate malnutrition (5–8 points), and severe malnutrition (9–12 points) (22). Subsequently, receiver operating characteristic (ROC) curve analysis was used to assess the ability of the ALI and CONUT scores (as continuous variables) to predict AF. The optimal cutoff values were determined based on the Youden index (specific values are provided in Supplementary Table 2). According to these cutoffs, both variables were transformed into binary categories: ALI (≤14.93 vs. >14.93) and CONUT score (≤4 vs. >4). To further explore the joint association of the ALI and CONUT scores with AF, patients were divided into four combined groups: (1) high ALI + low CONUT (ALI > 14.93 and CONUT ≤4); (2) high ALI + high CONUT (ALI > 14.93 and CONUT >4); (3) low ALI + low CONUT (ALI ≤ 14.93 and CONUT ≤4); and (4) low ALI + high CONUT (ALI ≤ 14.93 and CONUT >4). Additionally, to assess the robustness of the results, we reclassified the ALI and CONUT scores using alternative grouping methods (tertiles, mean, and median) to evaluate the consistency of the observed associations with AF.

### 2.4 Statistical analysis

All statistical analyses were performed using SPSS version 25.0 (IBM Corp., Armonk, NY, USA) and R software version 4.1.3 (R Foundation for Statistical Computing, Vienna, Austria). Missing covariates were handled using multiple imputation via the mice package in R. Normally distributed continuous variables were presented as mean ± standard deviation and compared using the Student’s t-test; non-normally distributed continuous variables were expressed as median (interquartile range) and compared using the Mann–Whitney U test or Kruskal-Wallis test, as appropriate; categorical variables were expressed as frequency (percentage) and compared using the chi-square test. All statistical tests were two-sided, and a *p*-value < 0.05 was considered statistically significant.

To evaluate the independent associations of the ALI and CONUT scores with AF, multivariate logistic regression models were constructed. Variables with a p-value < 0.05 in univariate analysis were initially selected, and variance inflation factors (VIFs) were calculated to assess multicollinearity, with variables exhibiting VIF ≥ 5 excluded. Three progressively adjusted models were developed: Model 1 was adjusted for age and sex; Model 2 was further adjusted for BMI, smoking history, drinking history, bronchial asthma, heart failure, hypertension, coronary artery disease, cerebrovascular disease, and pulmonary hypertension; Model 3 included all significant variables from the univariate analysis for full adjustment. Model 3 was constructed as a conservative adjustment model to assess whether the associations of ALI and CONUT with atrial fibrillation persisted after adjustment for established clinical risk factors and individual laboratory parameters. Given that albumin and blood cell indices are components of the composite scores, inclusion of these variables in the fully adjusted model was intended to evaluate the robustness and potential incremental association of ALI and CONUT beyond their individual components, rather than to imply causal independence.

Additionally, to further explore potential nonlinear relationships between the ALI and CONUT scores and AF risk, we employed restricted cubic spline (RCS) analysis to visualize dose–response associations. For variables demonstrating nonlinear trends, a two-piecewise logistic regression model was further applied to identify inflection points and segment effects. To further investigate the joint association of the ALI and CONUT score with AF, we constructed a combined exposure variable based on their optimal cutoff values, categorizing patients into four distinct groups. Using the “high ALI–low CONUT” group as the reference group, both univariate and multivariate logistic regression models were employed to evaluate the associations of the remaining combined groups with AF. The discriminative ability of each indicator for differentiating AF was evaluated using ROC curve analysis, and the DeLong test was used to compare the area under the curve (AUC) differences between models. The incremental discriminative ability from adding the ALI and CONUT scores was assessed using the AUC, net reclassification improvement (NRI), and integrated discrimination improvement (IDI). Model goodness-of-fit was evaluated using the Akaike Information Criterion (AIC) and likelihood ratio tests. Finally, the potential clinical utility of the models was assessed using decision curve analysis (DCA).

Subgroup analyses were conducted based on the following baseline characteristics: age (<75 / ≥75 years), sex, BMI (<18.5/18.5–23.9 / ≥24 kg/m^2^), smoking history, drinking history, heart failure, hypertension, diabetes, coronary artery disease, and pulmonary hypertension. These analyses were conducted using pre-defined categorical variables: (A) ALI group (with ALI > 14.93 as the reference); (B) CONUT group (with CONUT ≤4 as the reference); and (C) a combination of ALI and CONUT groups (with “ALI > 14.93 and CONUT ≤4” as the reference). Interaction effects between subgroup variables and exposure groups were assessed using interaction tests. A series of sensitivity analyses were performed to validate the robustness of the results, including: excluding patients with cerebrovascular disease, excluding patients on relevant medications, and using different variable grouping methods (tertiles, median, and mean).

## 3 Results

### 3.1 Baseline characteristics of the study population

This study included 1,510 patients with COPD, of whom 425 (28.15%) had comorbid AF. Based on the optimal ALI cutoff value of 14.93, determined by the Youden index in ROC analysis, patients were categorized into low and high ALI groups (Table 1). Compared to the high ALI group, patients with a low ALI were significantly older and had lower BMI, LVEF, RBC, PLT, L, ALB, UA, TC, and LDL-C levels, but higher WBC, N, AST, and BUN levels (all *p* < 0.05). The low ALI group also had a higher prevalence of heart failure, cerebrovascular disease, and pulmonary hypertension, but a lower prevalence of asthma. Regarding medications, aldosterone receptor antagonist use was more common, while beta-agonists and anticholinergic agents were less frequently used in the low ALI group. Most notably, the the proportion of patients with AF was significantly higher in the low ALI group.

**Table 1:** Baseline characteristics of the study population according to the ALI.

Characteristic	Overall (*n* = 1,510)	Low ALI (≤14.93) (*n* = 592)	High ALI (>14.93) (*n* = 918)	*p*-value
Age (years)	74 (68, 82)	77 (71, 83)	73 (66, 80)	**<0.001**
Male, *n* (%)	1,275 (84.44)	507 (85.64)	768 (83.66)	0.300
BMI (kg/m2)	21.5 (19.1, 24.1)	19.8 (17.9, 22.6)	22.3 (19.8, 24.8)	**<0.001**
LVEF (%)	64 (60, 68)	64 (58, 67)	65 (60, 68)	**<0.001**
Smoking, *n* (%)	873 (57.81)	350 (59.12)	523 (56.97)	0.409
Drinking, *n* (%)	539 (35.70)	215 (36.32)	324 (35.29)	0.685
Asthma, *n* (%)	36 (2.38)	7 (1.18)	29 (3.16)	**0.014**
Heart failure, *n* (%)	212 (14.04)	120 (20.27)	92 (10.02)	**<0.001**
Hypertension, *n* (%)	596 (39.47)	220 (37.16)	376 (40.96)	0.141
Diabetes, *n* (%)	217 (14.37)	84 (14.19)	133 (14.49)	0.872
Cerebrovascular disease, *n* (%)	144 (9.54)	73 (12.33)	71 (7.73)	**0.003**
Coronary heart disease, *n* (%)	235 (15.56)	102 (17.23)	133 (14.49)	0.151
Pulmonary hypertension, *n* (%)	610 (40.40)	289 (48.82)	321 (34.97)	**<0.001**
RBC (×10^9^/L)	4.22 (3.81, 4.63)	4.05 (3.63, 4.45)	4.35 (3.93, 4.73)	**<0.001**
WBC (×10^9^/L)	7.3 (5.7, 9.4)	8.7 (6.5, 11.3)	6.7 (5.2, 8.3)	**<0.001**
PLT (×10^9^/L)	206 (154, 261)	191 (143, 254)	214 (163, 264)	**<0.001**
N (×10^9^/L)	5.0 (3.6, 7.0)	7.1 (5.2, 9.6)	4.2 (3.2, 5.5)	**<0.001**
L (×10^9^/L)	1.24 (0.82, 1.70)	0.79 (0.54, 1.07)	1.54 (1.20, 1.96)	**<0.001**
M (×10^9^/L)	0.57 (0.40, 0.80)	0.59 (0.37, 0.87)	0.56 (0.42, 0.75)	0.373
ALT (U/L)	16 (12, 25)	17 (12, 27)	16 (12, 24)	0.383
AST (U/L)	23 (18, 30)	23 (18, 33)	22 (18, 29)	**0.038**
ALB (g/L)	36.3 (32.6, 39.4)	33.4 (30.2, 36.7)	37.9 (35.0, 40.6)	**<0.001**
BUN (mmol/L)	6.2 (4.8, 8.1)	6.8 (5.1, 9.3)	5.9 (4.7, 7.4)	**<0.001**
UA (μmol/L)	327 (257, 409)	300 (227, 402)	341 (272, 412)	**<0.001**
TC (mmol/L)	4.24 (3.46, 4.99)	3.95 (3.22, 4.74)	4.40 (3.64, 5.15)	**<0.001**
LDL-C (mmol/L)	2.35 (1.79, 2.98)	2.17 (1.66, 2.78)	2.50 (1.88, 3.12)	**<0.001**
HDL-C (mmol/L)	1.13 (0.92, 1.37)	1.14 (0.91, 1.39)	1.11 (0.93, 1.34)	0.667
Beta-agonists, n (%)	691 (45.76)	248 (41.89)	443 (48.26)	**0.015**
Anticholinergic agents, n (%)	768 (50.86)	257 (43.41)	511 (55.66)	**<0.001**
Aldosterone receptor antagonists, n (%)	304 (20.13)	152 (25.68)	152 (16.56)	**<0.001**
Metformin, *n* (%)	52 (3.44)	16 (2.70)	36 (3.92)	0.205
Beta-blockers, *n* (%)	276 (18.28)	113 (19.09)	163 (17.76)	0.513
Atrial fibrillation, *n* (%)	425 (28.15)	229 (38.68)	196 (21.35)	**<0.001**

*p* values in bold are < 0.05.

ALI, advanced lung cancer inflammation Index; BMI, body mass index; LVEF, left ventricle ejection fraction; RBC, red blood cell; WBC, white blood cell; PLT, platelet; N, neutrophil; L, lymphocyte; M, monocyte; ALT, alanine aminotransferase; AST, aspartate aminotransferase; ALB, albumin; BUN, blood urea nitrogen; UA, uric acid; TC, total cholesterol; LDL-C, low-density lipoprotein-cholesterol; HDL-C, high-density lipoprotein-cholesterol.

Similarly, using a CONUT cutoff value of 4, also derived from the Youden index, patients were stratified into two groups (Table 2). Patients in the high CONUT group were older and had higher N and BUN levels, but significantly lower BMI, LVEF, RBC, PLT, L, M, TC, LDL-C, and HDL-C levels (all *p* < 0.05). The prevalence of heart failure, cerebrovascular disease, coronary heart disease, and pulmonary hypertension was higher in this group, while the prevalence of asthma was lower. Pharmacologically, the high CONUT group showed higher utilization of aldosterone receptor antagonists and beta-blockers, but lower use of beta-agonists, anticholinergic agents, and metformin. Critically, AF was significantly more common in the high CONUT group.

**Table 2:** Baseline characteristics of the study population according to CONUT.

Characteristic	Overall (*n* = 1,510)	Low CONUT (≤4) (*n* = 1,019)	High CONUT (>4) (*n* = 491)	*p*-value
Age (years)	74 (68, 82)	72 (66, 79)	79 (72, 84)	**<0.001**
Male, *n* (%)	1,275 (84.44)	865 (84.89)	410 (83.50)	0.487
BMI (kg/m2)	21.5 (19.1, 24.1)	21.8 (19.4, 24.2)	20.7 (18.4, 23.5)	**<0.001**
LVEF (%)	64 (60, 68)	65 (60, 68)	63 (58, 67)	**<0.001**
Smoking, *n* (%)	873 (57.81)	597 (58.59)	276 (56.21)	0.381
Drinking, *n* (%)	539 (35.70)	368 (36.11)	171 (34.83)	0.625
Asthma, *n* (%)	36 (2.38)	34 (3.34)	2 (0.41)	**<0.001**
Heart failure, *n* (%)	212 (14.04)	100 (9.81)	112 (22.81)	**<0.001**
Hypertension, *n* (%)	596 (39.47)	402 (39.45)	194 (39.51)	0.982
Diabetes, *n* (%)				0.394
Cerebrovascular disease, *n* (%)	144 (9.54%)	83 (8.15%)	61 (12.42%)	**0.008**
Coronary heart disease, *n* (%)	235 (15.56)	140 (13.74)	95 (19.35)	**0.005**
Pulmonary hypertension, *n* (%)	610 (40.40)	356 (34.94)	254 (51.73)	**<0.001**
RBC (×10^9^/L)	4.22 (3.81, 4.63)	4.37 (3.98, 4.75)	3.90 (3.39, 4.30)	**<0.001**
WBC (×10^9^/L)	7.3 (5.7, 9.4)	7.2 (5.8, 9.2)	7.3 (5.2, 10.0)	0.665
PLT (×10^9^/L)	206 (154, 261)	217 (167, 267)	182 (129, 249)	**<0.001**
N (×10^9^/L)	5.0 (3.6, 7.0)	4.8 (3.6, 6.5)	5.7 (3.8, 8.3)	**<0.001**
L (×10^9^/L)	1.24 (0.82, 1.70)	1.47 (1.11, 1.89)	0.77 (0.54, 1.08)	**<0.001**
M (×10^9^/L)	0.57 (0.40, 0.80)	0.59 (0.43, 0.81)	0.53 (0.34, 0.77)	**<0.001**
ALT (U/L)	16 (12, 25)	17 (12, 25)	16 (11, 27)	0.171
AST (U/L)	23 (18, 30)	23 (18, 29)	23 (18, 33)	0.457
ALB (g/L)	36.3 (32.6, 39.4)	38.1 (35.9, 40.6)	31.5 (28.7, 33.7)	**<0.001**
BUN (mmol/L)	6.2 (4.8, 8.1)	6.0 (4.8, 7.5)	6.7 (5.1, 9.7)	**<0.001**
UA (μmol/L)	327 (257, 409)	330 (261, 406)	319 (244, 415)	0.280
TC (mmol/L)	4.24 (3.46, 4.99)	4.59 (3.93, 5.26)	3.42 (2.84, 4.12)	**<0.001**
LDL-C (mmol/L)	2.35 (1.79, 2.98)	2.61 (2.04, 3.17)	1.87 (1.41, 2.36)	**<0.001**
HDL-C (mmol/L)	1.13 (0.92, 1.37)	1.18 (0.97, 1.42)	1.00 (0.78, 1.24)	**<0.001**
Beta-agonists, *n* (%)	691 (45.76)	503 (49.36)	188 (38.29)	**<0.001**
Anticholinergic agents, *n* (%)	768 (50.86)	586 (57.51)	182 (37.07)	**<0.001**
Aldosterone receptor antagonists, *n* (%)	304 (20.13)	151 (14.82)	153 (31.16)	**<0.001**
Metformin, *n* (%)	52 (3.44)	42 (4.12)	10 (2.04)	**0.037**
Beta-blockers, *n* (%)	276 (18.28)	170 (16.68)	106 (21.59)	**0.021**
Atrial fibrillation, *n* (%)	425 (28.15)	213 (20.90)	212 (43.18)	**<0.001**

*p* values in bold are < 0.05.

CONUT, controlling nutritional status; BMI, body mass index; LVEF, left ventricle ejection fraction; RBC, red blood cell; WBC, white blood cell; PLT, platelet; N, neutrophil; L, lymphocyte; M, monocyte; ALT, alanine aminotransferase; AST, aspartate aminotransferase; ALB, albumin; BUN, blood urea nitrogen; UA, uric acid; TC, total cholesterol; LDL-C, low-density lipoprotein-cholesterol; HDL-C, high-density lipoprotein-cholesterol.

When comparing patients with and without AF, those with AF exhibited a more pronounced inflammatory and nutritional deficit profile: ALI values were significantly lower (14 [IQR 7–27] vs. 22 [IQR 11–34]; *p* < 0.001), with a higher proportion classified in the low ALI group (53.88% vs. 33.46%). Concurrently, CONUT scores were significantly higher (median 4 [IQR 3–6] vs. 3 [IQR 1–5]; *p* < 0.001), with nearly half (49.88%) of AF patients in the high CONUT group compared to only 25.71% of non-AF patients. Combined analysis further revealed that AF patients were predominantly concentrated in the “low ALI + high CONUT” subgroup (38.35% vs. 18.06%, p < 0.001), which represents the subgroup with the most severe compromise in both inflammatory and nutritional status. Notably, patients with AF did not exhibit a lower BMI compared with those without atrial fibrillation, complete baseline characteristics stratified by AF status are detailed in Supplementary Table 3.

### 3.2 Association of the ALI and CONUT with AF

Univariate logistic regression analysis identified multiple clinical variables significantly associated with AF (detailed results are provided in Supplementary Table S4). In univariate analysis, higher BMI was also associated with increased odds of atrial fibrillation (OR 1.049, 95% CI: 1.020–1.080). These significant variables were included in subsequent multivariate models to adjust for potential confounding factors. The analysis demonstrated that each one-unit increase in ALI (OR = 0.979, 95% CI: 0.972–0.986) or each one-point increase in CONUT score (OR = 1.267, 95% CI: 1.211–1.325) was significantly associated with AF risk (all *p* < 0.001). These associations remained statistically significant after multivariate adjustment. In the fully adjusted model (Model 3, Table 3), each unit increase in ALI as a continuous variable was associated with a 1.5% reduction in the odds of AF (OR = 0.985, 95% CI: 0.978–0.993, *p* < 0.001), while each point increase in CONUT score was associated with a 22.7% increase in the odds of AF (OR = 1.227, 95% CI: 1.133–1.330, p < 0.001). When analyzed as categorical variables, low ALI was significantly associated with higher odds of AF (OR = 0.505, 95% CI: 0.379–0.674, p < 0.001), whereas a high CONUT score was significantly associated with higher odds of AF (OR = 1.724, 95% CI: 1.300–2.286, p < 0.001). Trend analyses further revealed significant dose–response relationships: The odds of AF decreased across increasing ALI quartiles (*P* for trend = 0.001) and increased with more severe CONUT categories (*P* for trend < 0.001). Specifically, compared to the Q1 group, patients in the Q4 group had 54.2% lower odds of AF (OR = 0.458, 95% CI: 0.305–0.688), while those with severe malnutrition had 3.454-fold higher odds of AF compared to the normal nutrition group (OR = 4.454, 95% CI: 2.286–8.678).

**Table 3:** Multivariate logistic regression analysis for atrial fibrillation.

	Model 1		Model 2		Model 3	
Variables	OR (95% CI)	*P* value	OR (95% CI)	*P* value	OR (95% CI)	*p*-value
ALI						
Per unit increase	0.987 (0.980, 0.994)	**0.001**	0.984 (0.976, 0.992)	**<0.001**	0.985 (0.978, 0.993)	**<0.001**
Per SD increase	0.751 (0.638, 0.884)	**0.001**	0.697 (0.581, 0.838)	**<0.001**	0.721 (0.601, 0.865)	**<0.001**
ALI subgroup						
Low ALI (≤14.93)	Ref		Ref		Ref	
High ALI (>14.93)	0.537 (0.421, 0.687)	**<0.001**	0.483 (0.368, 0.635)	**<0.001**	0.505 (0.379, 0.674)	**<0.001**
ALI quartiles						
Q1	Ref		Ref		Ref	
Q2	0.593 (0.428, 0.820)	**0.002**	0.500 (0.354, 0.706)	**<0.001**	0.540 (0.375, 0.779)	**0.001**
Q3	0.455 (0.324, 0.639)	**<0.001**	0.425 (0.295, 0.611)	**<0.001**	0.442 (0.302, 0.649)	**<0.001**
Q4	0.553 (0.391, 0.783)	**<0.001**	0.449 (0.306, 0.661)	**<0.001**	0.458 (0.305, 0.688)	**<0.001**
*P* for trend		**0.001**		**<0.001**		**0.001**
CONUT						
Per unit increase	1.183 (1.127, 1.242)	**<0.001**	1.180 (1.120, 1.243)	**<0.001**	1.227 (1.133, 1.330)	**<0.001**
Per SD increase	1.546 (1.363, 1.754)	**<0.001**	1.537 (1.343, 1.758)	**<0.001**	1.702 (1.382, 2.097)	**<0.001**
CONUT subgroup						
Low CONUT (≤4)	Ref		Ref		Ref	
High CONUT (>4)	2.005 (1.559, 2.579)	**<0.001**	1.931 (1.478, 2.522)	**<0.001**	1.724 (1.300, 2.286)	**<0.001**
CONUT groups						
Normal	Ref		Ref		Ref	
Mild	2.344 (1.605, 3.421)	**<0.001**	2.373 (1.602, 3.514)	**<0.001**	2.043 (1.360, 3.071)	**0.001**
Moderate	3.569 (2.410, 5.286)	**<0.001**	3.428 (2.271, 5.174)	**<0.001**	2.742 (1.782, 4.219)	**<0.001**
Severe	4.649 (2.579, 8.381)	**<0.001**	5.134 (2.747, 9.594)	**<0.001**	4.454 (2.286, 8.678)	**<0.001**
*P* for trend		**<0.001**		**<0.001**		**<0.001**

Model 1: Adjusted for age and gender.

Model 2: Adjusted for age, gender, BMI, smoking, asthma, heart failure, hypertension, cerebrovascular disease, coronary heart disease, pulmonary hypertension.

Model 3: Adjusted for all the variables in Model 2 plus RBC, PLT, ALB, BUN, UA, LVEF.

OR, odds ratio; CI, confidence interval.

*p* values in bold are < 0.05.

### 3.3 Nonlinear association and threshold analysis

**Figure 2:**
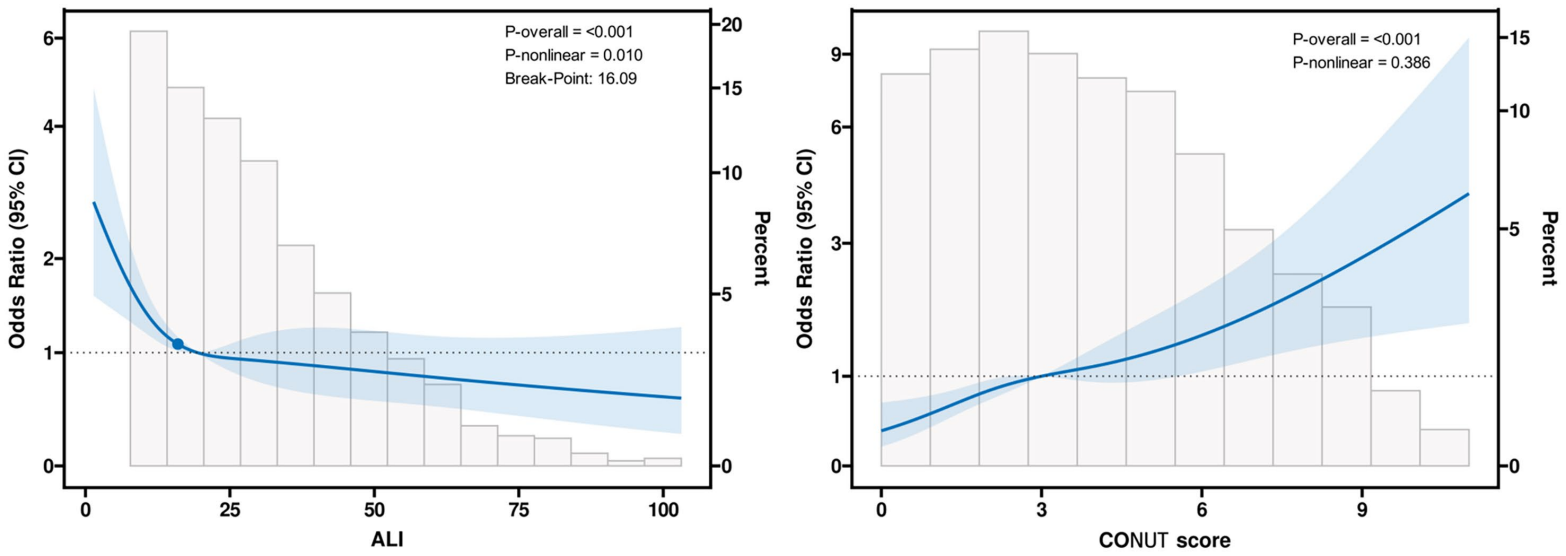
Nonlinear associations and threshold effects of ALI **(A)** and CONUT scores **(B)** with atrial fibrillation risk. The model was conducted with 4 knots at the 5th, 35th, 65th, and 95th percentiles (reference is the median). It was adjusted for age, gender, BMI, smoking, asthma, heart failure, hypertension, cerebrovascular disease, coronary heart disease, pulmonary hypertension, RBC, PLT, ALB, BUN, UA, LVEF. ALI, advanced lung cancer inflammation index; CONUT, controlling nutritional status; BMI, body mass index; RBC, red blood cell; PLT, platelet count; ALB, albumin; BUN, blood urea nitrogen; UA, uric acid; LVEF, left ventricular ejection fraction.

Restricted cubic spline analysis demonstrated a clear L-shaped relationship between the Advanced Lung Cancer Inflammation Index (ALI) and atrial fibrillation (AF), with a distinct threshold at approximately ALI = 16 (Figure 2A). When ALI values were below this level, the risk of AF increased sharply as ALI decreased. In contrast, once ALI exceeded this threshold, further increases in ALI did not result in additional reductions in AF risk, indicating that the protective effect of higher ALI levels reached a plateau. This threshold pattern was further supported by a two-piecewise logistic regression model, which showed that below the inflection point (ALI < 16.09), higher ALI levels were associated with progressively lower AF risk, whereas above this level (ALI ≥ 16.09), AF risk remained largely unchanged despite further increases in ALI (Table 4). By comparison, the CONUT score showed a consistent positive association with AF risk across its entire range, without evidence of a nonlinear pattern (Figure 2B).

**Table 4:** Results of two-piecewise logistic-regression model.

Analysis model	Adjusted OR (95% CI)	*p*-value
Linear logistic regression model	0.986 (0.978–0.995)	**0.002**
Two-piecewise logistic-regression model		
Inflection point(ALI)	16.090	
ALI < 16.090	0.930 (0.883–0.979)	**0.005**
ALI ≥ 16.090	0.995 (0.986–1.004)	0.270
*P* for likelihood test		**0.009**

Two-piecewise logistic-regression model was used to calculate the threshold effect of the ALI. If the likelihood test <0.05, it means the two-piecewise logistic regression model is superior to the single-line logistic regression model.

Adjusted for age, gender, BMI, smoking, asthma, heart failure, hypertension, cerebrovascular disease, coronary heart disease, pulmonary hypertension, RBC, PLT, ALB, BUN, UA, LVEF.

*p* values in bold are < 0.05.

### 3.4 Joint associations of ALI and CONUT with AF

To evaluate the combined influence of nutritional-inflammatory status on the risk of AF, patients were classified into four groups based on optimal cutoff values of the ALI (≤14.93 or >14.93) and CONUT score (≤4 or >4), using the group with ALI > 14.93 and CONUT ≤4 (representing the most favorable inflammatory-nutritional status) as the reference. As shown in Table 5, univariate analysis revealed that the other three groups all exhibited significantly elevated odds of AF. The group with ALI ≤ 14.93 and CONUT >4, representing the most compromised inflammatory-nutritional status, demonstrated the highest odds (OR = 3.615, 95% CI: 2.747–4.757, *p* < 0.001). After comprehensive multivariable adjustment, this group continued to show the highest odds of AF (adjusted OR = 2.420, 95% CI: 1.721–3.403, p < 0.001). Furthermore, the joint analysis indicated a significant trend effect (P for trend < 0.001), suggesting a graded increase in the odds of AF with decreasing ALI and increasing CONUT scores.

**Table 5:** Joint association of ALI and CONUT with atrial fibrillation.

ALI -CONUT combined groups	Univariate regression		Multivariate regression^a^	
	OR (95% CI)	*p*-value	Adjusted OR (95% CI)	*p*-value
ALI > 14.93 and CONUT ≤4	Ref		Ref	
ALI > 14.93 and CONUT >4	2.566 (1.727–3.813)	**<0.001**	1.156 (0.724–1.846)	0.543
ALI ≤ 14.93 and CONUT ≤4	1.718 (1.227–2.406)	**0.002**	1.530 (1.022–2.289)	**0.039**
ALI ≤ 14.93 and CONUT >4	3.615 (2.747–4.757)	**<0.001**	2.420 (1.721–3.403)	**<0.001**
*P* for trend		**<0.001**		**<0.001**

*p* values in bold are < 0.05.

^a^ Adjusted for age, gender, BMI, smoking, asthma, heart failure, hypertension, cerebrovascular disease, coronary heart disease, pulmonary hypertension, RBC, PLT, ALB, BUN, UA, LVEF.

### 3.5 Discriminative performance and incremental value

**Figure 3:**
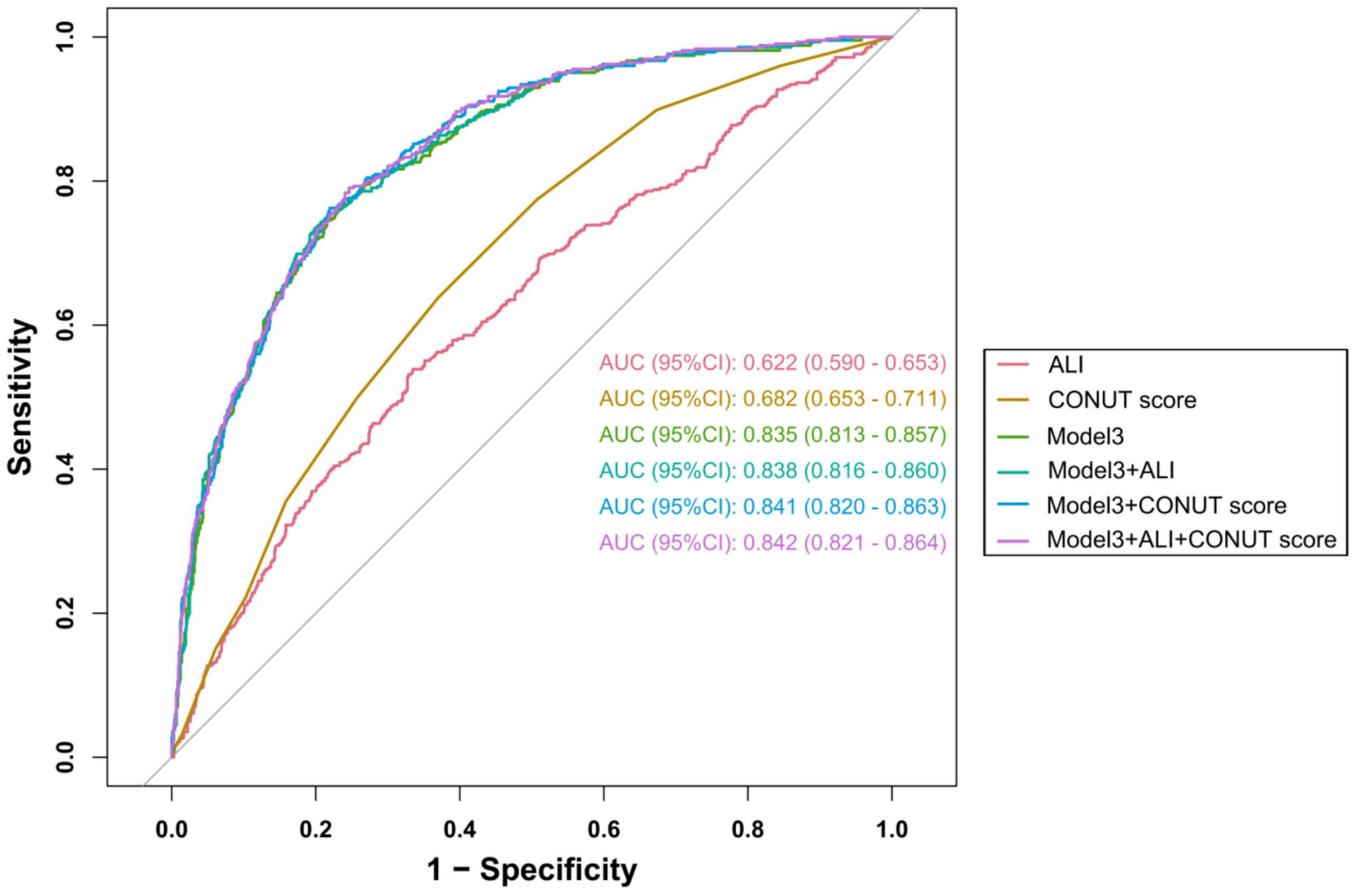
ROC analysis of atrial fibrillation prediction models: incremental value of ALI and CONUT scores. Model 3: adjusted for age, gender, BMI, smoking, asthma, heart failure, hypertension, cerebrovascular disease, coronary heart disease, pulmonary hypertension, RBC, PLT, ALB, BUN, UA, LVEF.

We systematically evaluated the improvement in model performance from adding the ALI and CONUT scores using ROC analysis, continuous NRI, IDI, and goodness-of-fit tests. As shown in Figure 3 and Table 6, the baseline model (Model 3) achieved an AUC of 0.835 (95% CI, 0.813–0.857). After the simultaneous inclusion of both the ALI and CONUT scores, model performance improved to an AUC of 0.842 (95% CI, 0.821–0.864, *p* = 0.031). Although the absolute increase in AUC was modest, this improvement—in conjunction with significant enhancements in NRI and IDI—collectively supports a clinically meaningful overall improvement in the model’s ability to discriminate between patients with and without AF. Specifically, compared to the baseline model, the combined model demonstrated significant reclassification improvement (NRI = 0.2732, 95% CI: 0.1551–0.3808, *p* < 0.001) and integrated discrimination improvement (IDI = 0.0150, 95% CI: 0.0073–0.0224, p < 0.001).

**Table 6:** Incremental value of adding ALI and CONUT for atrial fibrillation.

Prediction models	AUC (95% CI)	*p-value*	Continuous NRI (95% CI)	*p*-value	IDI (95% CI)	*p*-value
Model 3 without ALI and CONUT	0.835 (0.813–0.857)	**<0.001**	Reference		Reference	
Model 3 + ALI	0.838 (0.816–0.860)	0.214	0.1335 (0.0253–0.2424)	**0.022**	0.0069 (0.0019–0.0119)	**0.010**
Model 3 + CONUT	0.841 (0.820–0.863)	0.052	0.2561 (0.1431–0.3673)	**<0.001**	0.0128 (0.0058–0.0196)	**<0.001**
Model 3 + ALI + CONUT	0.842 (0.821–0.864)	**0.031**	0.2732 (0.1551–0.3808)	**<0.001**	0.0150 (0.0073–0.0224)	**<0.001**

NRI, net reclassification improvement; IDI, integrated discrimination improvement.

*p* values in bold are < 0.05.

**Figure 4:**
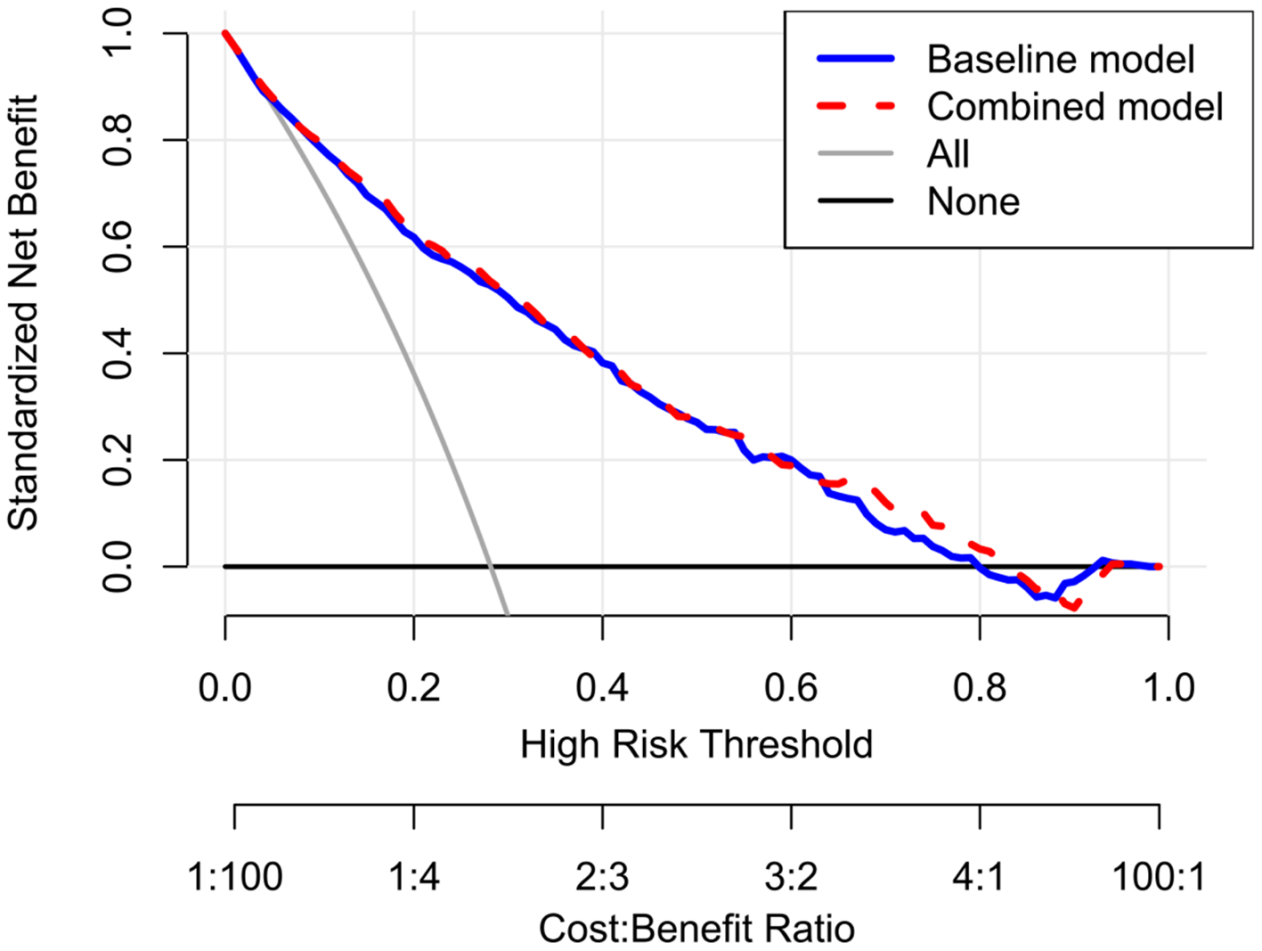
Decision curve analysis for atrial fibrillation risk: baseline model vs. combined model. Baseline model: Model3 (adjusted for age, gender, BMI, smoking, asthma, heart failure, hypertension, cerebrovascular disease, coronary heart disease, pulmonary hypertension, RBC, PLT, ALB, BUN, UA, LVEF). Combined model: Model3 + ALI + CONUT.

Goodness-of-fit assessments (Table 7) revealed a progressive decrease in the AIC value with the sequential addition of ALI, CONUT, and their combination (AIC of the combined model = 1392.91). All likelihood ratio tests were statistically significant (p < 0.001), indicating that the combined model provided the best fit. DCA further validated the clinical utility of the model (Figure 4). Across a wide range of threshold probabilities (baseline model: 0.01–0.79; combined model: 0.01–0.83), the combined model consistently yielded higher net clinical benefit than the baseline model, suggesting broader clinical applicability and improved utility in risk stratification and decision-making.

**Table 7:** Assessment of the goodness-of-fit of models.

Prediction models	AIC	LRT-*χ*^2^	df	*p*-value
Model 3 without ALI and CONUT	1426.23	Reference	Reference	Reference
Model 3 + ALI	1416.17	12.05	1	**<0.001**
Model 3 + CONUT	1393.96	34.27	1	**<0.001**
Model 3 + ALI + CONUT	1392.91	37.32	2	**<0.001**

AIC, Akaike information criterion; LRT, likelihood ratio test.

*p* values in bold are < 0.05.

### 3.6 Subgroup and sensitivity analyses

**Figure 5:**
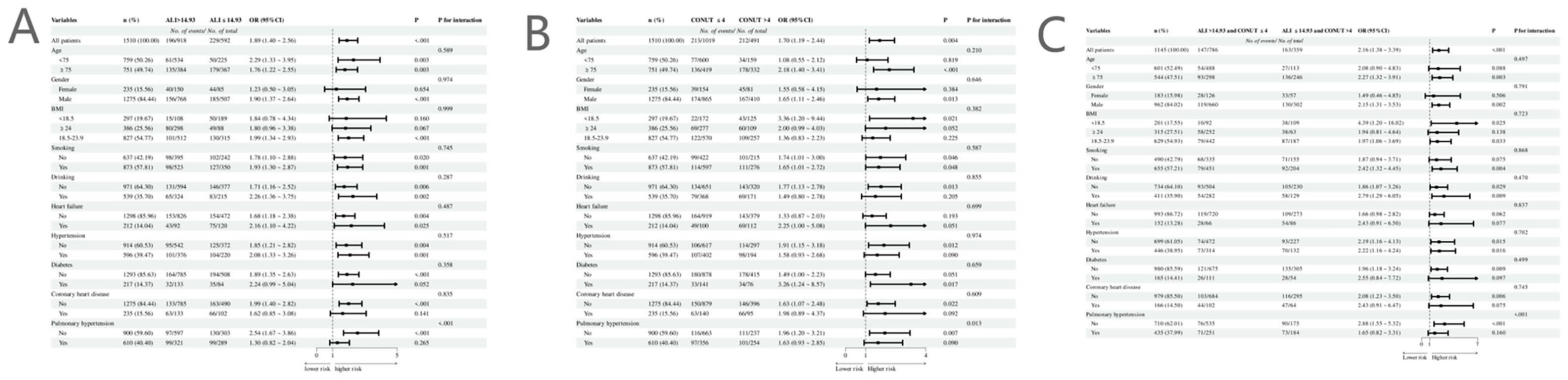
Subgroup analysis of atrial fibrillation risk by ALI and CONUT scores: individual and combined grouping. **(A)** ALI groups (high/low: 14.93). **(B)** CONUT groups (high/low: 4). **(C)** Combined groups (reference: ALI > 14.93 and CONUT≤4).

Subgroup analysis results (Figure 5) demonstrated that the associations between lower ALI and higher CONUT score with increased odds of AF were consistent across all subgroups stratified by age, sex, body mass index, smoking status, alcohol consumption, heart failure, hypertension, diabetes, and coronary heart disease (all interaction *p* > 0.05). However, significant interaction effects were observed specifically within the pulmonary hypertension subgroup for lower ALI, higher CONUT score, and their combined groupings (interaction *p* < 0.05). Specifically, among patients without pulmonary hypertension, both lower ALI (OR = 2.54, 95% CI: 1.67–3.86; *p* < 0.001) and higher CONUT score (OR = 1.96, 95% CI: 1.20–3.20; *p* = 0.007) were significantly associated with elevated odds of AF. Patients exhibiting both lower ALI and higher CONUT scores demonstrated the highest odds of AF (OR = 2.88, 95% CI: 1.55–5.32; p < 0.001). In contrast, these associations were not observed in patients with comorbid pulmonary hypertension.

To evaluate the robustness of the associations of ALI, CONUT score, and their combined groupings with AF, a series of sensitivity analyses were performed. First, in analyses excluding specific populations (Table 8)—namely, patients with cerebrovascular disease, or those administered metformin, *β*-blockers, aldosterone receptor antagonists, or β-agonists during hospitalization—the associations of lower ALI and higher CONUT score with increased odds of AF remained statistically significant (all *p* < 0.05). To mitigate potential bias introduced by classification methods, we further validated the robustness of the results using multiple grouping strategies (Table S5). Whether grouped by tertiles, median, mean, or inflection point, lower ALI levels and higher CONUT scores were consistently and significantly associated with increased odds of AF (all *p* < 0.05). Trend tests further supported an inverse correlation between ALI and AF prevalence, and a positive correlation between CONUT score and AF prevalence (*P* for trend < 0.001). These findings indicate that the associations of ALI and CONUT score with AF prevalence remain consistent across various exclusion criteria and grouping methods, confirming the robustness of the study results.

**Table 8:** Sensitivity analysis of the associations between atrial fibrillation and the ALI, the CONUT score, and their combined groupings.

Variables	OR (95% CI)				
	Analysis 1	Analysis 2	Analysis 3	Analysis 4	Analysis 5
ALI					
Per unit increase	0.983 (0.974, 0.991)***	0.986 (0.977, 0.994)*	0.984 (0.974, 0.994)*	0.989 (0.980, 0.998)*	0.985 (0.975, 0.995)*
Per SD increase	0.676 (0.555, 0.824)***	0.724 (0.602, 0.871)*	0.698 (0.554, 0.880)*	0.785 (0.641, 0.960)*	0.709 (0.566, 0.887)*
ALI subgroup					
Low ALI (≤14.93)	Ref	Ref	Ref	Ref	Ref
High ALI (>14.93)	0.470 (0.347, 0.638)***	0.513 (0.384, 0.687)***	0.450 (0.318, 0.636)***	0.490 (0.346, 0.695)***	0.481 (0.332, 0.696)***
ALI quartiles					
Q1	Ref	Ref	Ref	Ref	Ref
Q2	0.504 (0.341, 0.746)*	0.541 (0.374, 0.783)*	0.517 (0.334, 0.799)*	0.603 (0.385, 0.946)*	0.440 (0.275, 0.704)*
Q3	0.407 (0.271, 0.612)***	0.436 (0.296, 0.644)***	0.385 (0.241, 0.614)***	0.557 (0.356, 0.874)*	0.427 (0.260, 0.703)*
Q4	0.389 (0.252, 0.599)***	0.474 (0.314, 0.716)***	0.417 (0.255, 0.684)*	0.620 (0.389, 0.988)*	0.388 (0.229, 0.657)***
*P* for trend	***	*	*	***	*
CONUT					
Per unit increase	1.250 (1.147, 1.362)***	1.216 (1.121, 1.318)***	1.144 (1.073, 1.221)***	1.139 (1.067, 1.216)***	1.118 (1.044, 1.196)*
Per SD increase	1.784 (1.428, 2.228)***	1.661 (1.347, 2.048)***	1.419 (1.200, 1.679)***	1.402 (1.183, 1.662)***	1.334 (1.119, 1.591)*
CONUT subgroup					
Low CONUT (≤4)	Ref	Ref	Ref	Ref	Ref
High CONUT (>4)	1.640 (1.214, 2.216)*	1.654 (1.243, 2.201)*	1.655 (1.183, 2.317)*	1.537 (1.089, 2.171)*	1.641 (1.146, 2.350)*
CONUT groups					
Normal	Ref	Ref	Ref	Ref	Ref
Mild	2.026 (1.317, 3.115)*	2.056 (1.361, 3.105)*	1.852 (1.122, 3.058)*	1.702 (1.060, 2.734)*	1.932 (1.127, 3.313)*
Moderate	2.481 (1.573, 3.914)***	2.643 (1.709, 4.087)***	2.422 (1.437, 4.082)*	1.956 (1.176, 3.253)*	2.909 (1.477, 5.729)*
Severe	4.867 (2.390, 9.908)***	4.260 (2.174, 8.345)***	4.140 (1.950, 8.789)***	4.783 (2.207, 10.366)***	6.196 (2.085, 18.409)*
*P* for trend	***	***	***	***	*
ALI-CONUT combined groups					
ALI > 14.93 and CONUT≤4	Ref	Ref	Ref	Ref	Ref
ALI > 14.93 and CONUT > 4	1.108 (0.666, 1.843)	1.167 (0.729, 1.867)	1.027 (0.576, 1.830)	1.027 (0.554, 1.906)	1.235 (0.691, 2.207)
ALI ≤ 14.93 and CONUT≤4	1.813 (1.181, 2.785)*	1.587 (1.057, 2.383)*	1.738 (1.073, 2.817)*	1.711 (1.058, 2.767)*	1.777 (1.053, 2.999)*
ALI ≤ 14.93 and CONUT > 4	2.404 (1.678, 3.443)***	2.322 (1.644, 3.280)***	2.588 (1.719, 3.896)***	2.297 (1.521, 3.469)***	2.456 (1.582, 3.813)***
*P* for trend	***	***	***	***	***

Analysis 1: Excluding patients with cerebrovascular disease (*n* = 144).

Analysis 2: Excluding patients receiving metformin during hospitalization (*n* = 52).

Analysis 3: Excluding patients receiving beta-blocker therapy during hospitalization (*n* = 276).

Analysis 4: Excluding patients receiving aldosterone receptor antagonists during hospitalization (*n* = 304).

Analysis 5: Excluding patients receiving beta-agonist therapy during hospitalization (*n* = 691).

Adjusted for age, gender, BMI, smoking, asthma, heart failure, hypertension, cerebrovascular disease, coronary heart disease, pulmonary hypertension, RBC, PLT, ALB, BUN, UA, LVEF.

*p < 0.05; ***p < 0.001.

## 4 Discussion

This multicenter cross-sectional study demonstrates that both the Advanced Lung Cancer Inflammation Index (ALI) and the Controlling Nutritional Status (CONUT) score are independently associated with the prevalence of atrial fibrillation (AF) in patients with COPD. The two indices exhibited distinct relationship patterns with AF: ALI demonstrated a significant nonlinear L-shaped association characterized by a clear inflection point at 16.09, whereas the CONUT score showed a significant linear positive association. Furthermore, multivariate joint-effect analysis revealed that patients exhibiting both low ALI and high CONUT features had the highest odds of AF. This additive effect strongly suggests that the confluence of inflammatory activation and nutritional impairment creates a particularly vulnerable substrate for atrial fibrillation development in COPD patients. The integration of both scores into a baseline model yielded significant improvements in discriminative performance, reclassification capability, and potential clinical utility. These findings remained consistent across most predefined subgroups and were confirmed by sensitivity analyses, underscoring the robustness of our results. It is important to note that while ALI and CONUT share components (e.g., albumin), their integration of distinct parameters (BMI and NLR in ALI; cholesterol and lymphocytes in CONUT) suggests they may capture complementary aspects of the systemic pathophysiological burden. Our statistical models accounted for potential multicollinearity (VIF < 5), supporting their inclusion as independent variables.

Our study represents a significant conceptual shift from a unidimensional to a multidimensional assessment of AF risk in COPD. While conventional biomarkers like C-reactive protein (CRP) and albumin have well-established associations with AF risk in general populations (34–36), they provide limited insight into the complex, bidirectional pathophysiology characterizing COPD. The systemic manifestations of COPD extend far beyond pulmonary impairment, encompassing a self-perpetuating cycle of inflammation and catabolism that progressively erodes physiological reserve (20). In this context, composite indices like ALI and CONUT offer distinct advantages by capturing the integrated burden of this pathophysiological axis. The prognostic value of these composite indices has been demonstrated across diverse clinical contexts. ALI has emerged as a significant predictor of survival in non-small cell lung cancer, where it potentially reflects the balance between host immunity and tumor aggressiveness (37). Similarly, in heart failure populations, ALI has shown independent association with mortality, possibly capturing the intersection of systemic congestion, inflammation, and cardiac cachexia (38). The CONUT score, with its foundation in nutritional parameters with immune correlates, has demonstrated remarkable prognostic utility in conditions ranging from diabetic kidney disease to various malignancies (39–42). However, the specific relevance of these indices to AF in COPD—a condition characterized by a particularly pronounced inflammation-malnutrition axis—remained unexplored until now. Our findings address this critical knowledge gap by demonstrating that both ALI and CONUT are independently associated with AF prevalence in COPD patients, even after comprehensive adjustment for established cardiovascular risk factors. This suggests that these indices capture elements of AF pathophysiology not fully reflected by traditional risk models. Importantly, the retention of statistical significance after multivariable adjustment indicates that the inflammatory-nutritional axis represented by ALI and CONUT contributes to AF risk independently of other known risk factors in this population.

The associations observed in our study find strong support in established and emerging pathophysiological concepts. A low ALI, representing the confluence of neutrophilic inflammation (elevated NLR), depleted nutritional reserves (low albumin), and possible muscle wasting (low BMI), creates a milieu highly conducive to atrial fibrillation through multiple interconnected pathways. The inflammatory component contributes to atrial structural remodeling through fibroblast activation and collagen deposition, creating a fibrotic substrate that promotes reentrant circuits. Simultaneously, inflammatory mediators directly alter atrial electrophysiology by modulating ion channel function and reducing action potential duration (43–45). The oxidative stress associated with systemic inflammation further compounds electrical instability through lipid peroxidation and mitochondrial dysfunction (46, 47). Additionally, neurohormonal activation and autonomic imbalance, frequently present in inflammatory states, lower the threshold for triggered activity and facilitate AF initiation (48, 49). The nutritional dimensions captured by these indices similarly contribute to AF susceptibility. Hypoalbuminemia, reflecting both impaired synthesis and possible capillary leakage, reduces plasma oncotic pressure and may promote atrial wall edema and stretch (50, 51). Protein-energy malnutrition leads to muscle wasting that encompasses both skeletal and cardiac muscle, potentially resulting in atrial atrophy and fibrotic remodeling—a pattern consistent with the emerging concept of “atrial cardiomyopathy” (52). Electrolyte disturbances common in malnourished states, particularly hypokalemia and hypomagnesemia, create transient yet potent triggers for arrhythmogenesis (53, 54). Most importantly, our finding that patients with the combined phenotype of low ALI and high CONUT face the highest odds of AF provides strong clinical evidence for the “inflammation-malnutrition vicious cycle” in COPD-related AF. In this paradigm, persistent systemic inflammation accelerates protein catabolism through ubiquitin-proteasome and autophagy-lysosome pathways, while simultaneously suppressing appetite via cytokine-mediated effects (55, 56). The resulting nutritional impairment further amplifies inflammation through impaired antioxidant defense, gut barrier dysfunction, and altered body composition (57, 58). The co-existence of these two states creates a progressively worsening pathological environment that drives more pronounced atrial remodeling, ultimately explaining the substantially elevated AF risk observed in patients with this high-risk phenotype.

An important nuance of our findings is that patients with atrial fibrillation were not characterized by lower BMI, and higher BMI was in fact associated with increased odds of atrial fibrillation in univariate analysis. This observation highlights the limitation of BMI as a standalone indicator of nutritional or metabolic health in patients with COPD. BMI does not reflect systemic inflammation, body composition, or muscle mass, and higher BMI may coexist with significant muscle loss and metabolic dysfunction (59–61). In this context, the concept of sarcopenic obesity may help explain the apparent paradox whereby patients are not underweight yet exhibit pronounced inflammatory and nutritional impairment (62, 63). By integrating BMI with serum albumin and inflammatory burden, ALI appears better suited to capture these adverse states that may be masked by BMI alone (21, 64). In addition, obesity-related comorbidities, such as hypertension, diabetes, and obstructive sleep apnea may contribute to atrial fibrillation risk and should be considered when interpreting the relationship between BMI and atrial fibrillation in this population (65, 66).

Our findings establish readily calculable composite indices of inflammation and nutrition as clinically practical tools, indicating their potential for improving AF risk stratification within existing clinical frameworks. The specific cut-off values established in our study (ALI ≤ 14.93, CONUT >4) provide concrete thresholds that could inform screening intensity and prevention strategies, though these values require external validation before widespread implementation. The distinct association patterns we observed carry different clinical implications. The L-shaped relationship for ALI suggests the existence of a critical threshold below which AF risk increases dramatically. This pattern implies that interventions aimed at maintaining ALI above this threshold might be particularly beneficial for AF prevention. The linear relationship observed for CONUT indicates a more graded association, suggesting that even modest improvements in nutritional status might confer some benefit across the entire risk spectrum. The subgroup finding regarding pulmonary hypertension merits particular attention in clinical decision-making. The attenuation of the ALI/CONUT-AF association in PH patients suggests that in this subgroup, the potent arrhythmogenic effects of right heart pressure overload and associated neurohormonal activation may dominate AF pathogenesis (67–69). This observation implies that in COPD patients with significant pulmonary hypertension, the primary drivers of AF may shift from systemic inflammatory-nutritional pathways to the direct consequences of right ventricular pressure overload and atrial stretch. Consequently, while ALI and CONUT remain useful for most patients, AF risk assessment in this specific subgroup may require a greater focus on evaluating the severity of the underlying hemodynamic derangement. From a practical perspective, the combined assessment of ALI and CONUT appears to offer superior risk stratification compared to either index alone. The identification of patients with both low ALI and high CONUT scores could help target intensified monitoring strategies (e.g., extended Holter monitoring), which is crucial for capturing often-asymptomatic paroxysmal AF in this high-risk group. Additionally, these patients might represent candidates for more aggressive management of both inflammatory status and nutritional impairment, though the efficacy of such approaches for AF prevention specifically requires evaluation in intervention studies.

Several limitations must be acknowledged. First, the cross-sectional design precludes definitive conclusions regarding causal relationships or temporal sequence. While the observed associations are biologically plausible and statistically robust, we cannot exclude the possibility of reverse causation. Second, as a hospital-based study, our cohort likely represents patients with more severe disease, including a substantial proportion admitted for acute exacerbations of COPD, which may limit the generalizability of our findings to stable or community-based populations. Third, despite comprehensive adjustment for numerous potential confounders, residual confounding remains possible. Because this was a hospital-based study, a substantial proportion of patients were admitted during acute clinical states, including acute exacerbations of COPD or other acute illnesses. Acute inflammatory or infectious processes at hospital admission may influence inflammatory and nutritional biomarkers, such as ALI and CONUT. As detailed information on infection diagnoses or objective measures of acute illness severity was not systematically available, adjustment for acute exacerbation or infection severity was not feasible. Consequently, ALI and CONUT scores assessed at admission may, in some patients, reflect acute, transient inflammatory or nutritional changes rather than chronic baseline status, which could contribute to residual confounding. Furthermore, standardized indicators of COPD severity, including lung function parameters (e.g., FEV₁), GOLD stage, and prior exacerbation history, were not systematically available and therefore could not be incorporated into the analyses. As more severe COPD may be associated with both impaired nutritional/inflammatory status and a higher risk of atrial fibrillation through mechanisms such as hypoxemia, pulmonary hypertension, and systemic inflammation, the absence of these variables represents an additional source of potential residual confounding. In addition, factors such as physical activity levels, dietary patterns, socioeconomic status, and genetic predispositions, which were not systematically measured, could also influence both inflammatory/nutritional status and AF risk. Finally, while our study demonstrates significant associations between ALI/CONUT and AF prevalence, the incremental clinical value of these composite indices beyond established risk factors and simpler biomarkers remains to be fully established. Future studies directly comparing the predictive performance of these indices with conventional inflammatory and nutritional markers, as well as established clinical prediction tools, will be necessary to determine their optimal role in clinical risk stratification.

## 5 Conclusion

In conclusion, this study demonstrates that, among patients with chronic obstructive pulmonary disease, a lower Advanced Lung Cancer Inflammation Index (ALI) and a higher Controlling Nutritional Status (CONUT) score are independently associated with a higher prevalence of atrial fibrillation. These findings underscore the clinical relevance of the inflammation–nutrition axis in atrial fibrillation risk stratification among patients with COPD and suggest that ALI and CONUT, as readily available composite indices, may help identify individuals at elevated risk. If validated, these findings prompt further investigation into whether improving a patient’s nutritional or inflammatory status—through interventions such as dietary optimization, physical activity, or modulation of systemic inflammation—might help mitigate atrial fibrillation risk, representing a compelling hypothesis that warrants future prospective and interventional studies.

## Data Availability

The datasets analyzed during the current study are available from the corresponding author upon reasonable request. Requests to access these datasets should be directed to Hongjun Zhao, zhaohongjun@wmu.edu.cn.
