# Supplementary material for: Association of the advanced lung cancer inflammation index and controlling nutritional status score with atrial fibrillation in COPD patients: a multicenter cross-sectional study
Source: Front Nutr. 2026 Jan 27;13:1722288. doi: 10.3389/fnut.2026.1722288 (PMC12886012; doi:10.3389/fnut.2026.1722288)
Supplement: Supplementary file 1 [file Table_1.docx]

| **Table S1 CONUT Scoring Criteria for Nutritional Assessment** | | | | |
| --- | --- | --- | --- | --- |
| **Parameter** | **Score** | | | |
| Serum albumin (g/L) | ≥35 | 30-34.99 | 25-29.99 | <25 |
| Albumin score | 0 | 2 | 4 | 6 |
| Total cholesterol (mmol/L) | ≥4.66 | 3.63-4.65 | 2.59-3.62 | <2.59 |
| Cholesterol score | 0 | 1 | 2 | 3 |
| Total lymphocytes (10^9/L) | ≥1.6 | 1.2-1.59 | 0.8-1.19 | <0.8 |
| Lymphocyte score | 0 | 1 | 2 | 3 |

CONUT, controlling nutritional status.

| **Table S2 Optimal Cut-off Values of ALI and CONUT Score for Predicting Atrial Fibrillation Based on Youden Index** | | | | | |
| --- | --- | --- | --- | --- | --- |
| **Test** | **optimal cut-off value** | **Sensitivity** | **Specificity** | **Youden Index** | **AUC** |
| **ALI** | 14.93 | 0.539 | 0.665 | 0.204 | 0.622 |
| **CONUT** | 4 | 0.638 | 0.630 | 0.268 | 0.682 |

ALI, advanced lung cancer inflammation Index; CONUT, controlling nutritional status; AUC, area under the curve.

| **Table S3 Baseline demographic and clinical characteristics of the study population according to AF status** | | | | |
| --- | --- | --- | --- | --- |
| **Characteristic** | **Overall  (n=1510)** | **Non-AF  (n=1085)** | **AF  (n=425)** | ***p-value*** |
| Age (years) | 74 (68, 82) | 72 (65, 79) | 80 (74, 85) | **<0.001** |
| Male, n (%) | 1,275 (84.44) | 934 (86.08) | 341 (80.24) | **0.005** |
| BMI (kg/m2) | 21.5 (19.1, 24.1) | 21.3 (18.9, 23.9) | 22.0 (19.4, 24.6) | **0.001** |
| LVEF (%) | 64 (60, 68) | 65 (61, 68) | 60 (55, 66) | **<0.001** |
| Smoking, n (%) | 873 (57.81) | 648 (59.72) | 225 (52.94) | **0.016** |
| Drinking, n (%) | 539 (35.70) | 391 (36.04) | 148 (34.82) | 0.658 |
| Asthma, n (%) | 36 (2.38) | 34 (3.13) | 2 (0.47) | **0.002** |
| Heart failure, n (%) | 212 (14.04) | 94 (8.66) | 118 (27.76) | **<0.001** |
| Hypertension, n (%) | 596 (39.47) | 391 (36.04) | 205 (48.24) | **<0.001** |
| Diabetes, n (%) | 217 (14.37) | 150 (13.82) | 67 (15.76) | 0.334 |
| Cerebrovascular disease, n (%) | 144 (9.54) | 80 (7.37) | 64 (15.06) | **<0.001** |
| Coronary heart disease, n (%) | 235 (15.56) | 106 (9.77) | 129 (30.35) | **<0.001** |
| Pulmonary hypertension, n (%) | 610 (40.40) | 412 (37.97) | 198 (46.59) | **0.002** |
| RBC (×10⁹/L) | 4.22 (3.81, 4.63) | 4.29 (3.90, 4.68) | 4.03 (3.58, 4.49) | **<0.001** |
| WBC (×10⁹/L) | 7.3 (5.7, 9.4) | 7.3 (5.8, 9.3) | 7.0 (5.3, 9.4) | 0.130 |
| PLT (×10⁹/L) | 206 (154, 261) | 222 (175, 276) | 158 (116, 211) | **<0.001** |
| N (×10⁹/L) | 5.0 (3.6, 7.0) | 5.0 (3.6, 6.9) | 5.1 (3.6, 7.6) | 0.198 |
| L (×10⁹/L) | 1.24 (0.82, 1.70) | 1.34 (0.95, 1.79) | 0.95 (0.62, 1.39) | **<0.001** |
| M (×10⁹/L) | 0.57 (0.40, 0.80) | 0.58 (0.41, 0.81) | 0.56 (0.38, 0.76) | 0.068 |
| ALT (U/L) | 16 (12, 25) | 16 (12, 25) | 17 (12, 26) | 0.089 |
| AST (U/L) | 23 (18, 30) | 22 (18, 29) | 23 (18, 33) | 0.064 |
| ALB (g/L) | 36.3 (32.6, 39.4) | 36.8 (33.1, 40.1) | 35.2 (31.8, 37.9) | **<0.001** |
| BUN (mmol/L) | 6.2 (4.8, 8.1) | 5.9 (4.7, 7.5) | 7.2 (5.4, 9.5) | **<0.001** |
| UA (μmol/L) | 327 (257, 409) | 317 (252, 388) | 359 (266, 453) | **<0.001** |
| TC (mmol/L) | 4.24 (3.46, 4.99) | 4.46 (3.73, 5.15) | 3.62 (2.97, 4.37) | **<0.001** |
| LDL-C (mmol/L) | 2.35 (1.79, 2.98) | 2.50 (1.93, 3.11) | 1.99 (1.47, 2.61) | **<0.001** |
| HDL-C (mmol/L) | 1.13 (0.92, 1.37) | 1.13 (0.92, 1.37) | 1.12 (0.93, 1.35) | 0.620 |
| Beta-agonists, n (%) | 691 (45.76) | 562 (51.80) | 129 (30.35) | **<0.001** |
| Anticholinergic agents, n (%) | 768 (50.86) | 625 (57.60) | 143 (33.65) | **<0.001** |
| Aldosterone receptor antagonists, n (%) | 304 (20.13) | 105 (9.68) | 199 (46.82) | **<0.001** |
| Metformin, n (%) | 52 (3.44) | 41 (3.78) | 11 (2.59) | 0.254 |
| Beta-blockers, n (%) | 276 (18.28) | 116 (10.69) | 160 (37.65) | **<0.001** |
| ALI | 19 (10, 33) | 22 (11, 34) | 14 (7, 27) | **<0.001** |
| Standardized ALI | -0.23 (-0.66, 0.37) | -0.14 (-0.60, 0.44) | -0.48 (-0.79, 0.10) | **<0.001** |
| ALI subgroup, n (%) |  |  |  | **<0.001** |
| Low ALI (≤14.93) | 592 (39.21) | 363 (33.46) | 229 (53.88) |  |
| High ALI (>14.93) | 918 (60.79) | 722 (66.54) | 196 (46.12) |  |
| ALI quantile, n (%) |  |  |  | **<0.001** |
| Q1 | 376 (24.90) | 218 (20.09) | 158 (37.18) |  |
| Q2 | 379 (25.10) | 274 (25.25) | 105 (24.71) |  |
| Q3 | 376 (24.90) | 293 (27.00) | 83 (19.53) |  |
| Q4 | 379 (25.10) | 300 (27.65) | 79 (18.59) |  |
| CONUT | 3.00 (1.00, 5.00) | 3.00 (1.00, 5.00) | 4.00 (3.00, 6.00) | **<0.001** |
| Standardized CONUT | -0.18 (-0.96, 0.59) | -0.18 (-0.96, 0.59) | 0.20 (-0.18, 0.97) | **<0.001** |
| CONUT subgroup, n (%) |  |  |  | **<0.001** |
| Low CONUT (≤4) | 1,019 (67.48) | 806 (74.29) | 213 (50.12) |  |
| High CONUT (>4) | 491 (32.52) | 279 (25.71) | 212 (49.88) |  |
| CONUT groups, n (%) |  |  |  | **<0.001** |
| Normal | 397 (26.29) | 354 (32.63) | 43 (10.12) |  |
| Mild | 622 (41.19) | 452 (41.66) | 170 (40.00) |  |
| Moderate | 417 (27.62) | 241 (22.21) | 176 (41.41) |  |
| Severe | 74 (4.90) | 38 (3.50) | 36 (8.47) |  |
| ALI-CONUT combined groups, n (%) |  |  |  | **<0.001** |
| ALI >14.93 and CONUT≤4 | 786 (52.05) | 639 (58.89) | 147 (34.59) |  |
| ALI >14.93 and CONUT > 4 | 132 (8.74) | 83 (7.65) | 49 (11.53) |  |
| ALI≤14.93 and CONUT≤4 | 233 (15.43) | 167 (15.39) | 66 (15.53) |  |
| ALI≤14.93 and CONUT > 4 | 359 (23.77) | 196 (18.06) | 163 (38.35) |  |

*P* values in bold are < 0.05.

ALI, advanced lung cancer inflammation Index; CONUT, controlling nutritional status; BMI, body mass index; LVEF, left ventricle ejection fraction; RBC, red blood cell; WBC, white blood cell; PLT, platelet; N, neutrophil; L, lymphocyte; M, monocyte; ALT, alanine aminotransferase; AST, aspartate aminotransferase; ALB, albumin; BUN, blood urea nitrogen; UA, uric acid; TC, total cholesterol; LDL-C, low-density lipoprotein-cholesterol; HDL-C, high-density lipoprotein-cholesterol.

| **Table S4 Univariate logistic regression analysis for atrial fibrillation** | | | |
| --- | --- | --- | --- |
| **Variables** | **OR** | **95% CI** | **P value** |
| Age (years) | 1.103 | 1.087, 1.120 | **<0.001** |
| Male | 0.656 | 0.489, 0.881 | **0.005** |
| BMI (kg/m^2^) | 1.049 | 1.020, 1.080 | **<0.001** |
| LVEF (%) | 0.932 | 0.919, 0.946 | **<0.001** |
| Smoking | 0.759 | 0.605, 0.951 | **0.017** |
| Drinking | 0.948 | 0.750, 1.199 | 0.658 |
| Asthma | 0.146 | 0.035, 0.611 | **0.008** |
| Heart failure | 4.052 | 3.003, 5.468 | **<0.001** |
| Hypertension | 1.654 | 1.318, 2.076 | **<0.001** |
| Diabetes | 1.167 | 0.853, 1.595 | 0.334 |
| Cerebrovascular disease | 2.227 | 1.569, 3.160 | **<0.001** |
| Coronary heart disease | 4.025 | 3.018, 5.368 | **<0.001** |
| Pulmonary hypertension | 1.425 | 1.136, 1.787 | **0.002** |
| RBC (×10⁹/L) | 0.579 | 0.489, 0.687 | **<0.001** |
| WBC (×10⁹/L) | 0.996 | 0.965, 1.027 | 0.779 |
| PLT (×10⁹/L) | 0.990 | 0.988, 0.992 | **<0.001** |
| M (×10⁹/L) | 1.046 | 0.950, 1.152 | 0.360 |
| ALT (U/L) | 1.002 | 1.000, 1.004 | 0.129 |
| AST (U/L) | 1.001 | 0.999, 1.003 | 0.254 |
| ALB (g/L) | 0.945 | 0.924, 0.966 | **<0.001** |
| BUN (mmol/L) | 1.095 | 1.065, 1.125 | **<0.001** |
| UA (μmol/L) | 1.003 | 1.002, 1.004 | **<0.001** |
| LDL-C (mmol/L) | 0.993 | 0.955, 1.032 | 0.707 |
| HDL-C (mmol/L) | 1.005 | 0.981, 1.029 | 0.693 |
| ALI | 0.979 | 0.972, 0.986 | **<0.001** |
| ALI (Per SD) | 0.618 | 0.528, 0.725 | **<0.001** |
| ALI subgroup |  |  |  |
| ALI ≤ 14.93 | Ref |  |  |
| ALI > 14.93 | 0.430 | 0.342, 0.541 | **<0.001** |
| ALI Quartiles |  |  |  |
| Q1 | Ref |  |  |
| Q2 | 0.529 | 0.390, 0.717 | **<0.001** |
| Q3 | 0.391 | 0.284, 0.537 | **<0.001** |
| Q4 | 0.363 | 0.263, 0.501 | **<0.001** |
| CONUT | 1.267 | 1.211, 1.325 | **<0.001** |
| CONUT (Per SD) | 1.849 | 1.645, 2.078 | **<0.001** |
| CONUT subgroup |  |  |  |
| CONUT ≤ 4 | Ref |  |  |
| CONUT > 4 | 2.875 | 2.276, 3.633 | **<0.001** |
| CONUT group |  |  |  |
| Normal | Ref |  |  |
| Mild | 3.096 | 2.155, 4.448 | **<0.001** |
| Moderate | 6.012 | 4.147, 8.716 | **<0.001** |
| Severe | 7.799 | 4.478, 13.585 | **<0.001** |

*P* values in bold are < 0.05.

OR, odds ratio; CI, Confidence interval; BMI, body mass index; LVEF, left ventricle ejection fraction; RBC, red blood cell; WBC, white blood cell; PLT, platelet; M, monocyte; ALT, alanine aminotransferase; AST, aspartate aminotransferase; ALB, albumin; BUN, blood urea nitrogen; UA, uric acid; LDL-C, low-density lipoprotein-cholesterol; HDL-C, high-density lipoprotein-cholesterol; ALI, advanced lung cancer inflammation Index; CONUT, controlling nutritional status; SD, standard deviation.

| **Table S5** **Sensitivity analysis assessing the robustness of the associations of ALI, CONUT with atrial fibrillation.** | | |
| --- | --- | --- |
| **Variables** | **Adjusted OR (95% CI)** | ***P-value*** |
| ALI |  |  |
| By tertiles |  |  |
| T1 | Ref |  |
| T2 | 0.545 (0.394, 0.755) | **<0.001** |
| T3 | 0.444 (0.310, 0.636) | **<0.001** |
| P for trend |  | **<0.001** |
| By median split |  |  |
| Low ALI (≤19.41) | Ref |  |
| High ALI (>19.41) | 0.608 (0.456, 0.809) | **0.001** |
| By mean split |  |  |
| Low ALI (≤24.54) | Ref |  |
| High ALI (>24.54) | 0.565 (0.417, 0.767) | **<0.001** |
| By inflection point |  |  |
| Low ALI (≤16.09) | Ref |  |
| High ALI (>16.09) | 0.529 (0.397, 0.704) | **<0.001** |
| CONUT |  |  |
| By tertiles |  |  |
| T1 | Ref |  |
| T2 | 1.988 (1.297, 3.049) | **0.002** |
| T3 | 2.689 (1.790, 4.041) | **<0.001** |
| P for trend |  | **<0.001** |
| By median split |  |  |
| Low CONUT (≤3) | Ref |  |
| High CONUT (>3) | 1.825 (1.347, 2.474) | **<0.001** |
| By mean split |  |  |
| Low CONUT (≤3.48) | Ref |  |
| High CONUT (>3.48) | 1.712 (1.293, 2.266) | **<0.001** |

Adjusted for age, gender, BMI, smoking, asthma, heart failure, hypertension, cerebrovascular disease, coronary heart disease, pulmonary hypertension, RBC, PLT, ALB, BUN, UA, LVEF.

OR, odds ratio; CI, confidence interval; ALI, advanced lung cancer inflammation Index; CONUT, controlling nutritional status; BMI, body mass index; RBC, red blood cell; PLT, platelet count; ALB, albumin; BUN, blood urea nitrogen; UA, uric acid; LVEF, left ventricular ejection fraction

*P* values in bold are < 0.05
